# Stability Studies of Kynurenine Pathway Metabolites in Blood Components Define Optimal Blood Processing Conditions

**DOI:** 10.1177/11786469231213521

**Published:** 2023-12-15

**Authors:** Benjamin Heng, Ananda Staats Pires, Sharron Chow, Shivani Krishnamurthy, Brooke Bonnell, Sonia Bustamante, Gilles J Guillemin

**Affiliations:** 1 Macquarie Medical School, Faculty of Medicine and Health Sciences, Macquarie University, Sydney, NSW, Australia; 2 Bioanalytical Mass Spectrometry Facility, University of New South Wales, Sydney, Australia

**Keywords:** Biochemistry, tryptophan, kynurenine pathway, serotonin, analytical chemistry

## Abstract

The kynurenine pathway (KP) is the main pathway of tryptophan (TRP) metabolism that generates energy for multiple cellular processes. The activity of this pathway has been shown to be dysregulated in multiple human diseases. The resultant modulation of metabolites has been suggested to comprise biomarkers to track disease progression or could identify new therapeutic targets. While metabolite changes can be measured readily in blood, there is limited knowledge on the effect of blood matrices and sample processing time may have on the stability of KP metabolites. Understanding the stability of KP metabolites in blood is integral to obtaining accurate KP data to correlate with clinical pathology. Hence, the aim of this study was to assess the concentration of KP metabolites in matched whole blood, plasma and serum. The impact of pre-analytical sample processing time in the various blood matrices was also analysed. Serum and plasma had the higher concentration of KP metabolites compared to whole blood. Furthermore, concentrations of KP metabolites declined when the collected blood was processed after 24 hours storage at 4°C. Our study shows that that type of blood matrix and the time to processing have an impact on the stability of the KP metabolites. Serum or plasma are the preferred choice of matrix and the isolation of these matrices from whole blood is best performed immediately after collection for optimal analytical KP data.

## Introduction

**Figure 1.**
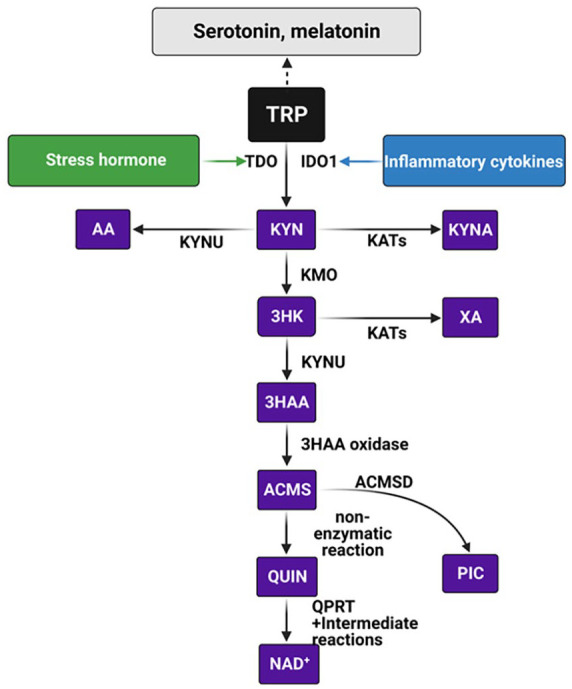
Schematic representation of kynurenine pathway metabolites production. Tryptophan (TRP; highlighted in black) is a precursor for serotonin and melatonin pathway (highlighted in grey), however, the majority of the TRP is catabolized through the kynurenine pathway (KP; KP metabolites are highlighted in purple). Tryptophan 2,3-dioxygenase (TDO), one of the rate-limiting enzymes of the kynurenine pathway, is activated by elevated stress hormone (highlighted in green). Indoleamine 2,3-dioxygenase (IDO), another rate-limiting enzyme of the kynurenine pathway is activated in the presence of inflammatory mediators (highlighted in blue). Once the KP is activated, TRP is metabolized into a series of intermediates through specific enzymes catalysing each reaction in various cells/tissues. Kynurenine (KYN), a central metabolite of the KP and can be catabolized into 3 different intermediates, 3-hydroxykynurenine (3HK), kynurenic acid (KYNA) and anthranilic acid (AA) by kynurenine monooxygenase (KMO), kynurenine aminotransferase (KATs) or kynureninase (KYNU) respectively. 3HK is further converted to 3-hydroxyanthranilic acid (3HAA) by KYNU and then to 2-amino-3-carboxymuconate semialdehyde (ACMS) by 3HAA oxydase. Alternatively, 3HK can also be metabolized by KATs into xanthurenic acid (XA). The metabolite ACMS is positioned at a key junction of the pathway to either form the metabolite quinolinic acid (QUIN), a precursor for de novo synthesis of NAD^+^ or converted to picolinic acid (PIC) by 2-amino-3-carboxymuconate semialdehyde decarboxylase (ACMSD).

Tryptophan (TRP) is one of the essential amino acids that has been widely studied due to its involvement in human physiological and pathological processes. Among the pathways involved in TRP metabolism, the kynurenine pathway (KP) has received the most attention because it catabolizes over 90% of TRP (Figure 1). The main physiological function of the KP is to generate nicotinamide adenine dinucleotide (NAD^+^), an important enzyme co-factor for cellular energy production.^1^ While KP metabolism is tightly regulated in normal physiological conditions, during infection, inflammation and/or stress the KP can be shunted towards the production of several bioactive metabolites which mediate disease progression. Indeed, KP dysregulation has been reported in a wide range of neurological diseases, psychiatric disorders, immune dysregulation and cancers.^2-4^

The first bioactive metabolite, KYN, is a key modulator of the immune system and is produced by the enzymes indolamine 2,3-dioxygenase 1 (IDO1) or tryptophan 2,3-dioxygenase (TDO). KYN is strongly associated with suppression of antitumour immunity^3^ and is the precursor for the downstream metabolites kynurenic acid (KYNA), anthranilic acid (AA) and 3-hydroxylkynurenine (3HK) by the activity of 3 different downstream enzymes in separate sub-branches of the KP. As KYNA is an endogenous N-methyl-D-aspartate (NMDA) receptor antagonist, KYNA mediates neuroprotection which has been demonstrated in animal models of neurodegenerative diseases.^5^ 3HK is a free radical generator which can trigger oxidative damage and cell death.^6^ 3HK also leads to quinolinic acid (QUIN) which is a neurotoxic, gliotoxic, proinflammatory metabolite by binding to the NMDA receptor and inducing excitotoxicity, Ca2+ influx and oxidative stress.^7^ In the central nervous system, QUIN has been shown to accumulate and mediates neuronal death in neurodegenerative diseases.^8,9^ Under normal physiological condition, QUIN is usually catabolize into NAD^+^. The metabolite picolinic acid (PIC) has neuroprotective properties possibly by binding metal ions and modulating cellular ROS.^10^ Another metabolite, xanthurenic acid (XA), can also be derived from 3HK. XA has been shown to have strong affinity to G-protein-coupled receptors. Its interactions with these receptors on brain cells have been observed to impact the dopaminergic activity and cationic channels.^11,12^ Considering the strong correlation between KP dysregulation and neurodegenerative/neuropsychiatric disease, the accurate quantitative measurement of KP metabolites in human fluids and tissue has emerged as a key challenge.

The area of research that has received intense attention is the performance of different analytical methods to quantify KP metabolites. These methods originally involved high-performance liquid chromatography (HPLC) and recently, more sensitive techniques including liquid or gas chromatography coupled with mass spectrometry (MS), tandem MS/MS and high-resolution MS.^13-15^ Advances in technology and analytical chemistry have led to novel highly sensitive and optimized protocols to overcome difficulties in analysing the KP metabolites in complex sample matrices as detailed in a recent review by Mrštná et al.^16^ However, the impact of quality control surrounding storage or processing conditions has been less well studied and may, at least in part, explain why studies of the KP in the same disease often produce contradictory results such as suicide^17,18^ and schizophrenia.^19,20^

Human whole blood, serum and plasma are commonly used matrices in clinical and biological studies. However, different collection techniques and the coagulation cascade impact concentrations of metabolites in these matrices.^21^ Additionally, the choice of the sample matrix has been shown to either enhance or suppress the ion intensity of an analyte, and impact the reproducibility and accuracy of the assay, alternatively known as the matrix effect.^22,23^ The effect of different sample matrices on KP metabolite concentration have not been fully characterized and compared. Additionally, there are multiple pre-analytical factors related to the processing of laboratory specimens that can contribute to result variation and/or inaccuracy.^24^ Frequently in clinical and biological studies, blood samples are collected outside the laboratory and transported for later processing and testing. Accordingly, delay in centrifugation and storage of specimens at incorrect temperatures can also influence concentrations of metabolites in sample matrices.^25^ Therefore, in the present study a metabolomics analysis of KP metabolites was performed comparing whole blood, plasma and serum samples from the same individual. Additionally, different pre-analytical sample processing and handling procedures that is, time of storage, centrifugation status and choice of buffer for metabolite extraction, was performed and impact on KP metabolite concentrations was assessed.

## Materials and Methods

### Reagents, standards and chromatography consumables

KP standards were purchased from Sigma Aldrich (Sydney, Australia). Isotopically enriched internal standard (IS) for KP metabolites were purchased from Toronto Research Chemicals (Toronto, Canada). LCMS grade acetonitrile was purchased from Honeywell Company (Germany). LCMS grade formic acid was purchased from Fisher Chemical (United States). MilliQ water used in the LC analysis was prepared using Milli-Q^®^ IQ 7000 Water Purification (Germany). Phenex™-RC 4 mm Syringe Filters with 0.2 μm membrane were purchased from Phenomenex (United States).

### Participants recruitment

Healthy volunteer participants (n = 12) were recruited by Macquarie University Neuroinflammation Research Group (Sydney, NSW, Australia) between April 2022 and July 2022. The study included both male (n = 5; 41.7%) and female (n = 7; 58.3%) healthy volunteers aged 26 to 45 years old. None of the participants experienced any inflammatory disease or other health symptoms for 2 weeks prior to sample collection. This study was approved by the Macquarie University Human Ethics Committee (HREC 520211087035353). The participation in the study was on a completely voluntary basis, and all participants signed informed consent forms.

### Blood collection, separation and storage

**Figure 2.**
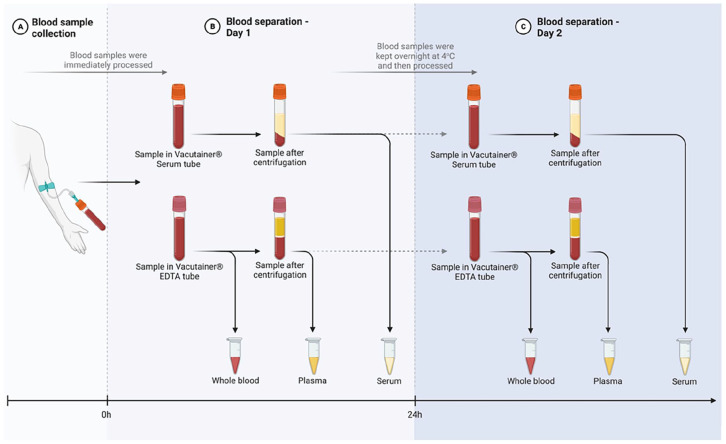
Experimental design. Blood samples were collected and separated into 3 different matrices, whole blood, plasma and serum, at 2 different time points, 0 hour (Day 1) and 24 hours (Day 2). (A) Blood samples were taken by venipuncture from the median cubital vein fossa into 2 tubes: (i) a 10 mL vacutainer^®^ EDTA collection tube and (ii) a 10 mL vacutainer^®^ Serum collection tube. (B) An aliquot of the EDTA-whole blood was taken and stored immediately at −80°C until further analysis. The samples in the vacutainer^®^ EDTA and vacutainer^®^ Serum tubes were then centrifuged and the supernatant containing plasma and serum, respectively, were storage immediately at −80°C until further analysis (Day 1). (C) The remain samples in the vacutainer^®^ EDTA and vacutainer^®^ Serum tubes were then gently mixed by inversion and stored at 4°C overnight for an identical separation process 24 hours later (Day 2).

Fasted blood samples were taken by venepuncture from the median cubital vein fossa by a trained phlebotomist, into one 10 mL vacutainer^®^ EDTA collection tube and one 10 mL vacutainer^®^ Serum collection tube (Becton Dickenson) (Figure 2A). The collected blood samples were divided into whole blood, plasma and serum aliquots at 2 different time points, 0 and 24 hours. First, an aliquot of the EDTA-whole blood was taken and stored immediately at −80°C until further analysis. The samples in the vacutainer^®^ EDTA and vacutainer^®^ serum tubes were then centrifuged at 800×*g* and 2500×*g* for 15 minutes respectively. An aliquot of the supernatant containing plasma or serum was then collected and processed as described below before storing at −80°C until further analysis (Figure 2B). The remaining samples in the vacutainer^®^ EDTA and vacutainer^®^ Serum tubes were then gently mixed by inversion and stored at 4°C overnight for an identical separation process 24 hours later (Figure 2C).

### Sample and standard preparation for liquid chromatography

Aliquots of whole blood, plasma and serum (150 μL) were deproteinized by adding 1 volume (150 μL) of 10% (w/v) trichloroacetic acid (TCA). The mixture was kept at 4°C for 10 minutes, vortexed and then centrifuged at 12 000×*g* at 4°C for 10 minutes. The supernatants were filtered through a 0.22 μm polytetrafluoroethylene syringe filter (Millex, Merck-Millipore, CA, USA), transferred into glass vials and capped prior to analysis.

Each metabolite was prepared at 10 mM concentration and stored at −80°C. Intermediate standards were prepared by diluting from the 10 mM stock and combining all compounds stock solutions to yield 100, 10 and 1 μM concentration, which were prepared fresh on the same day as analysis. Calibrating solutions (0, 5, 10, 20, 50, 100 and 200 nM for KYNA, 3HK, 3HAA and AA; 0, 0.5, 1, 2, 5, 10 and 20 μM for KYN; 0, 5, 10, 20, 50, 100 and 200 μM for TRP) were prepared fresh on the same day as analysis by dilution with MilliQ water, prior to transfer into glass vials and capped.

### Sample and standard preparation for gas chromatography

An aliquot (50 μL) of already deproteinized whole blood, plasma or serum was mixed with a fixed volume of deuterated internal standards for QUIN and PIC (d3-QUIN and d4-PIC, respectively), dried under vacuum and derivatized with 120 μL of trifluoroacetic anhydride and 1,1,1,3,3,3 hexafluoroisopropanol at a 1:1 ratio for 1 hour at 60°C. Fluorinated esters were isolated with toluene before being washed with 5% sodium bicarbonate. The upper organic layer was collected and washed with 1 mL of MilliQ water and dried using sodium sulphate packed pipette tips. Finally, the samples were transferred into glass vials prior to analysis.

Stock solutions of the internal standards d3-QUIN and d4-PIC were prepared at 10 mM concentration in deuterium oxide and stored at −80°C. Intermediate standards were prepared by diluting from 10 mM stock and combined with the 2 internal standards to yield 1 μM concentration. Calibrating solutions (0, 10, 20, 50, 100 and 200 nM) were prepared fresh in the same day of analysis by mixing aliquots of intermediate standards with a fixed volume of internal standard mixture in new tubes, vacuum dried and derivatized in an identical manner as described in the sample preparation for gas chromatography.

### Quantification of KP metabolites by ultra-high performance liquid chromatography (uHPLC) and HPLC

uHPLC analysis of TRP, KYN, 3-HK, 3-HAA and AA was carried out using a uHPLC system (Agilent 1290 Infinity, CA, USA) that contains an auto-sampler (4°C), a temperature-controlled column compartment (38°C), fluorescence detector (G1321B; xenon flash lamp; Agilent) and diode array (G4212A; Agilent). The analysis method was carried out as previously described.^26^ The standards and samples (20 µL) were injected into an analytical column ZORBAX Rapid Resolution High Definition C18 (2.1 mm × 150 mm, 1.8 μm particle size; Agilent, CA, USA) by reverse phase gradient elution at 38°C. The mobile phase used was 0.1 M sodium acetate at pH 4.6 and the assay and was run with an isocratic flow rate of 0.75 mL/minute for 12 minutes. The fluorescence detector was set at excitation/emission wavelength of 280 nm/438 nm for detection of TRP and 320 nm/438 nm for detection of 3HAA and AA. The UV detector was set at 365 nm (reference signal off) to detect KYN and 3HK. The chromatogram output of these KP metabolites was analysed using the Agilent OpenLAB CDS ChemStation (Edition C.01.04). The quantification of these KP metabolites was performed by interpolation using a calibration curve and the results were expressed as μmol/L or nmol/L. Excel 2016 was used for regression analysis of the results obtained.

HPLC analysis of KYNA were conducted using a HPLC system (Agilent 1260 Infinity, Agilent, CA, USA) that includes an auto-sampler (4°C), a temperature-controlled column compartment (30°C) and a fluorescence detector (G1321B xenon flash lamp, Agilent, CA, USA). The analysis method was carried out as previously described.^26^ Chromatographic separation was achieved by injection of standards and samples (10 μL) onto an analytical column ZORBAX Rapid Resolution High Definition C18 (4.6 mm × 100 mm, 3.5 μm particle size, Agilent Technologies, CA, USA) using a reverse phase gradient elution at 30°C. The mobile phase used was 95% of 0.05 M sodium acetate and 0.05 M zinc acetate, pH 5.2 and 5% HPLC grade acetonitrile. The assay was run with an isocratic flow rate of 1 mL/minute for 8 minutes. KYNA was detected with the fluorescence detector at an excitation/emission wavelength of 344 nm/388 nm. The chromatogram output of KYNA was analysed using the Agilent OpenLAB CDS ChemStation (Edition C.01.04). The quantification of KYNA was performed by interpolation using a calibration curve and the results were expressed as nmol/L.

### Quantification of metabolites by Gas Chromatography/Mass Spectrometry (GC/MS)

GC/MS analysis of QUIN and PIC was performed using an Agilent 7890 gas chromatograph coupled with an Agilent 5975 mass spectrometer following a protocol previously described.^26^ The standards and samples (1 µL) were injected using spitless mode onto a HP-5MS GC capillary column (Agilent, CA, USA). Analysis was carried out in negative chemical ionization mode. Selected ions (m/z 273 for PIC, m/z 277 for d4-PIC, m/z 467 for QUIN and m/z 470 for d3-QUIN) were simultaneously monitored. GC oven was held at 75°C for 3 minutes and then ramped to 300°C at a rate of 25°C/minute and held at 300°C for 4 minutes for a total run time of 15.6 minutes. Calibration curves of QUIN and PIC were constructed using peak area ratios (peak area of the QUIN and PIC divided by peak area of d3-QUIN and d4-PIC, respectively) of each calibrating solution versus its concentration. The concentrations of QUIN and PIC in the samples were obtained from these calibration curves. All spectra were processed, and peak areas integrated using Agilent GC/MSD ChemStation software (Edition 02.02.1431). Levels of QUIN and PIC were calculated and expressed as nmol/L.

### Sample and standard preparation for Liquid Chromatography with tandem mass spectrometry (LC-MS/MS)

An aliquot of whole blood, plasma or serum (200 μL) was deproteinized by adding 100% methanol (800 μL) for a final mixture of 80% methanol. The mixture was kept at 4°C for 10 minutes, vortexed and centrifugated at 12 000×*g* at 4°C for 10 minutes. The supernatant was then transferred to a new tube and mixed with a fixed volume of deuterated internal standards and vacuum dried at room temperature. Next, the sample was reconstituted with 100 μL of MilliQ water and filtered through a 0.22 μm Phenex™-RC 4 mm Syringe Filters (Phenomenex, USA) into a glass vial and capped prior to LC-MS/MS analysis.

10 mM concentration of each metabolite was prepared in MilliQ water and stored at −80°C. Stock solutions of internal standards were prepared at 10 mM concentration in deuterium oxide and stored at −80°C. Intermediate standards were prepared by diluting the stock solutions and combined to yield 100 and 10 μM concentrations. Calibrating solutions for metabolites in serotonin pathway were prepared at 0, 5, 10, 20, 40, 60, 80, 100, 200, 400 and 600 nM and for KP metabolites, 3HK, 3HAA, AA, XA and KYNA: 0, 20, 40, 60, 80, 100, 200 nM; KYN: 0, 100, 200, 300, 400, 1000, 2000, 3000, 4000 nM; TRP: 0, 20, 20, 30 and 40 μM). All calibrating solutions were prepared fresh on the same day of analysis by combining a fixed volume of internal standard mixture with aliquots of intermediate standards in new tubes, vacuum dried, reconstituted in 100 μL of MilliQ water and transferred into glass vials prior to LC-MS/MS analysis.

### Quantification of metabolites by LC-MS/MS

LC-MS/MS analysis of TRP, KYN, 3HK, 3HAA, 5-hydroxytryptophan (5HTP), xanthurenic acid (XA), 5-hydroxyindoleacetic acid (5HIAA), serotonin, KYNA and AA was carried out using a TSQ Vantage mass spectrometer (Thermofisher, USA) connected to a Vanquish solvent delivery/autosampler system (Thermo-Dionex, USA), according to a protocol previously described.^27^ Briefly, chromatographic separation was achieved by injecting standards and samples (20 μL) onto a Luna^®^ PFP(2)100 Å reversed phase column (2 × 150 mm, 3 μm; Phenomenex) with the temperature of the column compartment set at room temperature by reverse phase gradient elution at 25°C. A binary solvent gradient was used which consisted of aqueous 0.1% formic acid (A) and methanol (B) at a flow rate of 300 μL/minute. The gradient elution was set to start at 10% buffer B for 2 minutes, ramped to 60% buffer B at 4 minutes, then to 100% buffer B at 8 minutes. At 14.4 minutes set to 10% B and equilibrated for 5.6 minutes. Total run time was 20 minutes. Mass spectrometric detection was performed using multiple reaction monitoring (MRM) with heated electrospray ionization (HESI) source in positive mode. MSD parameters and MRM transitions used for individual metabolites and labelled metabolites were set as previously described.^27^

Calibration curves of individual metabolites were constructed using the peak area ratios (peak area of the metabolite divided by peak area of the selected IS) of each calibrating solution versus its concentration. These calibration curves were used to calculate the concentrations of the endogenous metabolites in the samples. All spectra and peak areas were processed using XcaliburTM software (version 2.2, 2011, ThermoFisher Scientific, Waltham, MA) while automated data processing was performed using the LCQuan feature of the software. Levels were calculated and expressed as μmol/L or nmol/L.

### Statistical analysis

Initial analysis of the data were carried out to screen for the presence of multiple outliers using ROUT test and all the outliers were excluded. Subsequently, the data were analysed for normality using the Kolmogorov-Smirnov normality test. Two-way analysis of variance (ANOVA) was used with time and matrix as fixed factors to test for statistically significant time-matrix effect, but also time or matrix effects separately. Multiple comparisons of time-matrix groups were performed using Sidak post hoc analysis, wherein comparation between different matrices was performed when significant matrix effect was observed and/or comparation between Day 1 and Day 2 was performed when a significant time effect was present. An adjusted *P* ⩽ .05 was considered significant for all analyses. All statistical analysis was carried out with GraphPad Prism Software (Prism 9, GraphPad Software Inc.).

## Results

### Demographics

The demographic data are provided in Table 1. Overall, 58.3% (n = 7) of the cohort were women while 41.7% (n = 5) were men. The mean age was 32.5 years old, ranging from 26 to 45 years old.

**Table 1.** Demographics measures in healthy volunteers.

	Healthy volunteers (n = 12)
Sex (female)	(n = 7) 58.3%
Age (y)	32.5 [26-45]
Age range distribution (y)	
20-29	(n = 3) 25%
30-39	(n = 8) 66.7%
40-49	(n = 1) 8.3%

Sex and age distribution are expressed in percentage (%); Age is expressed as mean [range].

### KP metabolite profile in whole blood, plasma and serum measured by HPLC

**Figure 3.**
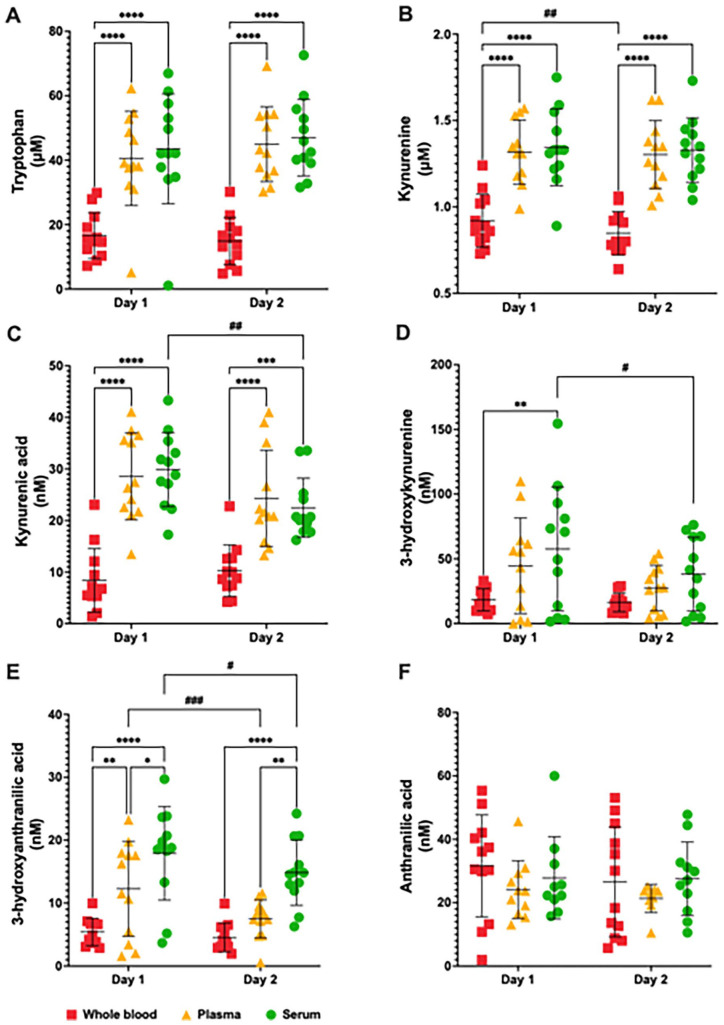
Kynurenine pathway metabolite profile measured by high-performance liquid chromatography in whole blood, plasma and serum from healthy volunteers at day 1 and day 2. Levels of (A) tryptophan, (B) kynurenine, (C) kynurenic acid, (D) 3-hydroxykynurenine, (E) 3-hydroxyanthranilic acid and (F) anthranilic acid in the whole blood (red squares), plasma (yellow triangles) and serum (green circles) at day 1 and day 2. *P < .05, **P < .01, ***P < .001, ****P < .0001, 2-way ANOVA test followed by the post hoc test of Sidak for multiple comparations of Matrix (Whole blood vs Plasma vs Serum). ^#^*P* < .05, ^##^*P* < .01, ^###^*P* < .001, 2-way ANOVA test followed by the post hoc test of Sidak for multiple comparations of Time (Day 1 vs Day 2). Abbreviations: ANOVA, Analysis of variance.

To understand the time and matrix effect on the concentration of KP metabolites, we compared the results of whole blood, plasma and serum from healthy volunteers processed immediately (Day 1) and 24 hours after sample collection (Day 2) (Figure 2). Our HPLC measurements of the KP metabolite profile in whole blood, plasma serum at Day 1 and 2 showed various significant changes (Figure 3). Specifically, we observed a significant matrix effect on the levels of TRP [*F*(2, 33) = 28.58, *P* < .001], KYN [*F*(2, 33) = 25.29, *P* < .001], 3HK [*F*(2, 33) = 4.275, *P* < .05] and 3HAA [*F*(2, 32) = 16.24, *P* < .001]. Notably, TRP and KYN levels were higher in plasma and in serum compared to the whole blood on both Day 1 and Day 2 (TRP, Figure 3A; KYN, Figure 3B). A significant time-matrix effect was observed for KYNA [*F*(2, 33) = 6.016, *P* < .001], which measured higher levels in plasma and serum when compared to the whole blood on both Day 1 and at the Day 2 (Figure 3C). Furthermore, 3HK levels were higher in serum when compared to the whole blood at Day 1 (Figure 3D), while 3HAA levels were higher in plasma and serum compared to the whole blood on both Day 1 and at the Day 2. Additionally, 3HAA levels were higher in the serum in comparison to plasma at Day 1 (Figure 3E).

We also noted a significant time effect on the levels of KYN [*F*(1, 32) = 7.401, *P* < .05], 3HK [*F*(1, 32) = 8.284, *P* < .01] and 3HAA [*F*(1, 32) = 19.29, *P* < .001]. KYN levels were lower in whole blood at Day 2 compared to Day 1 (Figure 3B), while 3HK levels were lower in serum at Day 2 compared to Day 1 (Figure 3D). KYNA and 3HAA levels were lower in plasma and serum on Day 2 compared to Day 1 (Figure 3C and E). Although 2-way ANOVA reveal a significant time effect on the AA levels [*F*(1, 28) = 4.231, *P* < .05], the post hoc analysis showed no significant differences in AA levels between Day 1 and Day 2. Complete KP metabolite profile measured by HPLC and statistical analysis for multiple comparisons can be found in the supplementary information (Table S1).

### KP metabolite profile in whole blood, plasma and serum measured by GC/MS

**Figure 4.**
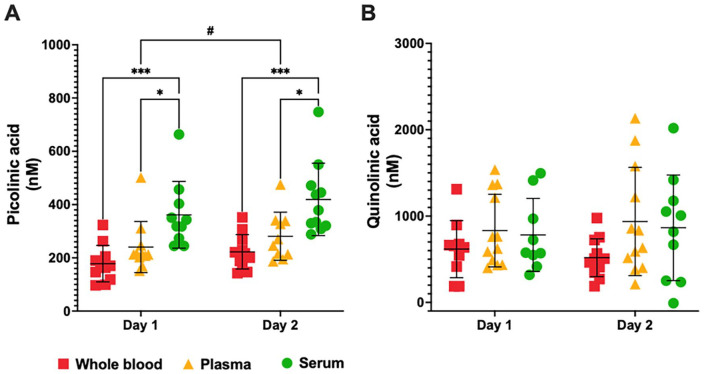
Kynurenine pathway metabolite profile measured by gas chromatography/mass spectrometry in whole blood, plasma and serum from healthy volunteers at day 1 and day 2. Levels of (A) picolinic acid and (B) quinolinic acid in the whole blood (red squares), plasma (yellow triangles) and serum (green circles) at day 1 and day 2. *P < .05, ***P < .001, 2-way ANOVA test followed by the post hoc test of Sidak for multiple comparations of Matrix (Whole blood vs Plasma vs Serum). ^#^*P* < .05, 2-way ANOVA test followed by the post hoc test of Sidak for multiple comparations of Time (Day 1 vs Day 2).

We also observed a significant matrix effect [*F*(2, 30) = 11.21, *P* < .001] and time effect [*F*(1, 28) = 16.40, *P* < .001] on PIC concentration. PIC levels were higher in plasma and serum when compared to whole blood at Day 1 and at Day 2. Additionally, PIC levels were higher in the plasma on Day 2 compared to Day 1 (Figure 4A). No significant time or matrix effects were observed for QUIN levels (Figure 4B). Complete KP metabolite profile measured by GC/MS and statistical analysis of multiple comparisons can be found in the supplementary information (Table S2).

### Temporal KP metabolite profile in whole blood, plasma and serum measured by LC-MS/MS

**Figure 5.**
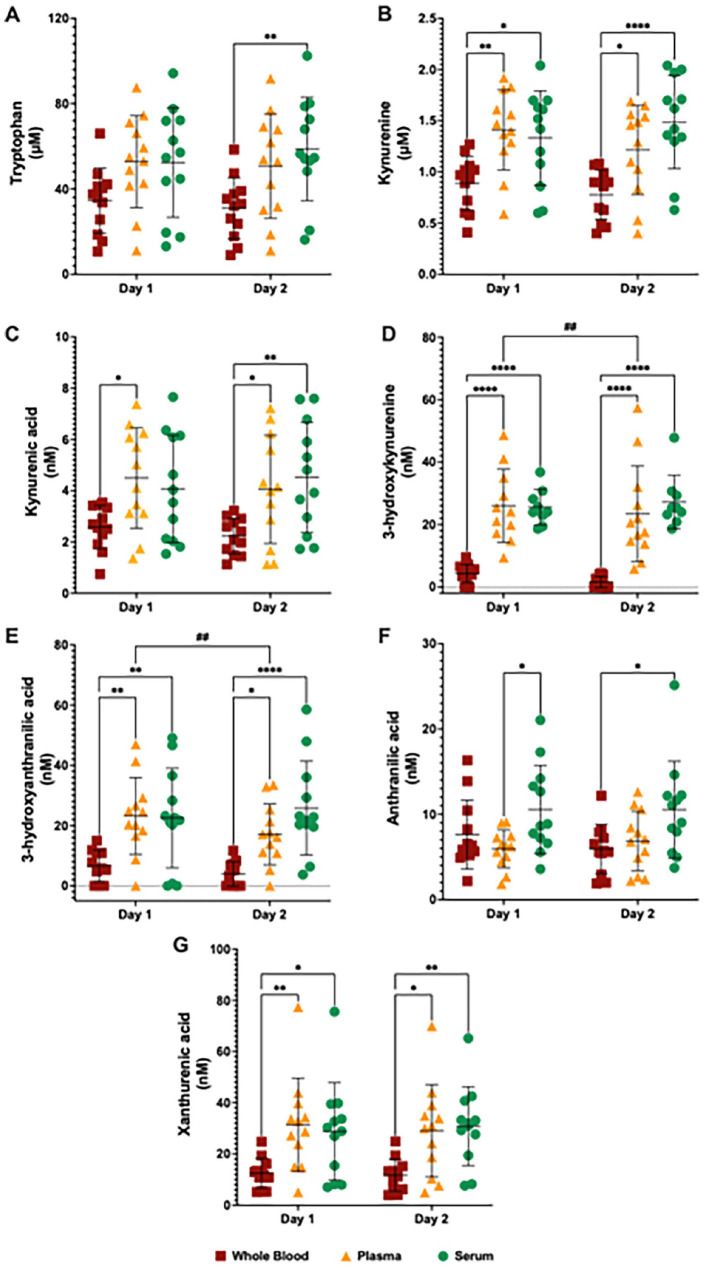
Kynurenine pathway metabolite profile measured by liquid chromatography with tandem mass spectrometry in whole blood, plasma and serum from healthy volunteers at day 1 and day 2. Levels of (A) tryptophan, (B) kynurenine, (C) kynurenic acid, (D) 3-hydroxykynurenine, (E) 3-hydroxyanthranilic acid, (F) anthranilic acid and (G) xanthurenic acid in the whole blood (dark red squares), plasma (dark yellow triangles) and serum (dark green circles) at day 1 and day 2. *P < .05, **P < .01, ****P < .0001, 2-way ANOVA test followed by the post hoc test of Sidak for multiple comparations of Matrix (Whole blood vs Plasma vs Serum). ^##^*P* < .01, 2-way ANOVA test followed by the post hoc test of Sidak for multiple comparations of Time (Day 1 vs Day 2). Abbreviation: ANOVA: Analysis of variance.

In our assessment of the KP metabolite profile using LC-MS.MS, there was a significant time-matrix effect on the levels of KYN [*F*(2, 33) = 4.225, *P* < .05], 3HK [*F*(2, 28) = 3.858, *P* < .05] and 3HAA [*F*(2, 32) = 6.546, *P* < .05]. In particular, KYN and 3HAA levels were higher in plasma and serum when compared to whole blood at Day 1 and at the Day 2, while no difference in KYN and 3HAA levels between Day 1 and Day 2 were found (KYN, Figure 5B; 3HAA, Figure 5E). 3HK levels were higher in plasma and in serum when compared to whole blood at Day 1 and at Day 2, while 3HK levels were lower in the plasma at Day 2 compared to Day 1 (Figure 5D).

Furthermore, there was a significant matrix effect on the levels of TRP [*F*(2, 33) = 4.157, *P* < .05], KYNA [*F*(2, 33) = 5.324, *P* < .01], AA [*F*(2, 33) = 4.277, *P* < .05] and XA [*F*(2, 33) = 6.020, *P* < .05]. TRP levels were higher in serum and plasma when compared to whole blood at Day 1 (Figure 5A). KYNA levels were higher in plasma when compared to whole blood at Day 1, while on Day 2, KYNA levels were higher in serum and plasma when compared to the whole blood at Day 2 (Figure 5C). AA levels were higher in serum when compared to plasma at Day 1, whereas AA levels were higher in serum when compared to whole blood at Day 2 (Figure 5F). Additionally, XA levels were higher in plasma and in serum when compared to whole blood at Day 1 and at the Day 2 (Figure 5G). Complete KP metabolite profile measured by LC-MS/MS and statistical analysis for multiple comparisons can be found in the supplementary information (Table S3).

### Concentrations of serotonin metabolites were higher in serum and plasma

**Figure 6.**
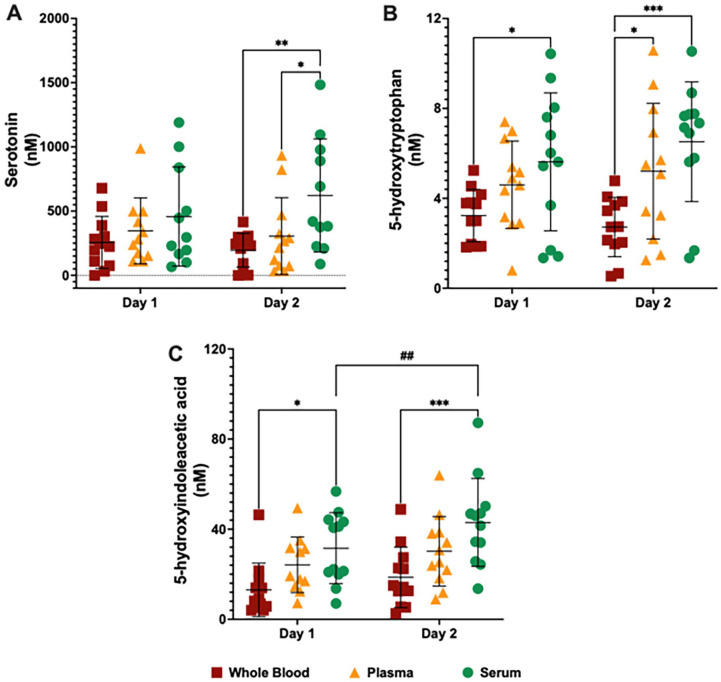
Serotonin-related metabolite profile measured by liquid chromatography with tandem mass spectrometry in whole blood, plasma and serum from healthy volunteers at day 1 and day 2. Levels of (A) serotonin, (B) 5-hydroxytryptophan and (C) 5-hydroxyindoleacetic acid in the whole blood (dark red squares), plasma (dark yellow triangles) and serum (dark green circles) at day 1 and day 2. *P < .05, **P < .01, ***P < .001, 2-way ANOVA test followed by the post hoc test of Sidak for multiple comparations of Matrix (Whole blood vs Plasma vs Serum). ^##^*P* < .01, 2-way ANOVA test followed by the post hoc test of Sidak for multiple comparations of Time (Day 1 vs Day 2). Abbreviation: ANOVA: Analysis of variance.

Considering that TRP is also the substrate for the serotonin pathway, we analysed concentrations of the serotonin metabolites 5HTP, serotonin and 5HIAA in all 3 matrices using LC-MS/MS (Figure 6), assessing time and matrix effects. There was a significant time-matrix effect on the levels of [*F*(2, 31) = 7.016, *P* < .01], which were higher in plasma and in serum when compared to whole blood at Day 2, while no significant difference in serotonin levels between Day 1 and Day 2 was found (Figure 6A).

There was a significant matrix effect on the levels of 5HTP [*F*(2, 33) = 6.437, *P* < .01] and 5HIAA [*F*(2, 33) = 7.231, *P* < .01]. 5HT levels were higher in serum when compared to whole blood at Day 1, while 5HTP levels were higher in serum and plasma compared to whole blood at Day 2 (Figure 6B). 5HIAA levels were higher in serum when compared to whole blood at Day 1 and at Day 2 (Figure 6C). Finally, there was a significant time effect on the levels of 5HIAA [*F*(1, 32) = 16.28, *P* < .001], which were lower in serum at Day 1 compared to Day 2 (Figure 6C). Complete serotonin metabolite profile measured by LC-MS/MS and statistical analysis for multiple comparisons can be found in the supplementary information (Table S4).

## Discussion

The KP is the main pathway of TRP metabolism to catabolize TRP to NAD^+^. In addition to its primary function, it also generates several bioactive metabolites that have redox, immune suppressive activity, neurotoxic or neuroprotective effect. Considering the high number of biological pathways and processes that the KP can modulate, the activity of the KP has gained substantial interest as a potential marker to track disease progression, assess severity or identify potential new therapeutic targets. This has seen a concomitant development of assays to monitor KP enzymes and metabolites. Considering that blood collection from patients or healthy participants is much easier and less invasive, it is the matrix of choice for metabolic assessment.^28^ However, there is no consensus on which blood component is ideal for the assessment of the KP and whether technical challenges such as sample processing time can impact the stability of the metabolites. In addition, the period in which blood should be analysed following sample collection must also be considered, due to (i) the ability of the different blood cell types, including peripheral blood mononuclear cells, platelets and red blood cells, to catabolize TRP and generate KP metabolites; (ii) the stability of the KP metabolites overtime, especially when biological fluid cannot be process immediately after collection due to logistical reasons and (iii) low physiological concentration. These factors are more likely to affect the levels of highly redox-active KP metabolites such as 3HK, 3HAA and QUIN.^6,29^ Hence, this study assessed the differences of KP metabolites concentration between various blood components and at different processing time. Our results indicate that serum and plasma are generally better blood components than the whole blood to quantify most of the KP metabolites. This is supported by significantly higher concentration of KP metabolites detected in these 2 different matrices as compared to whole blood in all 3 analytical chemistry techniques used. Additionally, the metabolites KYN, KYNA, 3HK and 3HAA analysed in this study using HPLC were present in lower concentration in the samples that were processed 24 hours after collection when compared to the samples that were immediately analysed. In the analysis of samples using LC/MS-MS, concentration of 3HK and 5HIAA were lower in 24 hours old samples.

That higher concentrations of the KP metabolites could be detected in serum and plasma as compared to whole blood could be due to a combination of factors. Firstly, it could be partially due to the volume displacement effect from the isolation step of serum and plasma.^30^ Serum and plasma are obtained after centrifugation to remove cellular components of the blood, which concentrates low molecular weight KP metabolites in these blood components as the sample effectively shrinks when bulky cell matter is removed. Another potential explanation for the different KP metabolite concentrations in each blood component matrix could also be due, in part, to incomplete dissociation of KP metabolites from albumin during protein precipitation using 10% TCA or 100% methanol to remove interfering proteins prior to chromatography and MS analysis respectively.^31,32^ Although all samples were treated equally, the presence of cellular components in whole blood may have reduce the availability of TCA and methanol from disassociating albumin-bound KP metabolites.^33^ Finally, the third potential factor could be enhanced matrix effects from the presence of undetected matrix components in whole blood that may reduce or suppress the ion intensity of the analytes.^22,23^

Interestingly, an earlier study detected higher concentrations of TRP in whole blood as compared to serum.^34^ They concluded that the increased concentrations were due to a combination of the haemolysis of red blood cells and time taken to process the samples.^34^ The concentration of TRP reported in whole blood and serum were similar at 26.5 and 22.7 mM, respectively, while whole blood used in our study had significantly lower concentration compared to serum and plasma. The difference in concentration of metabolites could be due to the different quantification methods used in each study. In their study, nuclear magnetic resonance spectrometry was employed that only is capable of semi-quantifying the metabolites and was limited by the annotation/identification of specific metabolites.^35,36^ Our study used a targeted approach with metabolite annotation that allows higher sensitivity and sensibility for quantification and identification of a specific metabolite.^35,37^ As noted above, the other potential contributing factor could be the time duration before sample processing prior to analysis. All the samples in this study were processed at the same time while Stringer et al allowed the whole blood samples to incubate at room temperature during the isolation of serum before processing together. This extra incubation time could have allowed metabolically active cellular components to produce similar concentration of metabolites as the serum.

It is also worth noting that there was a noticeable higher level of most of the KP metabolites in serum compared to plasma. One plausible explanation for this difference could be attributed to the presence of EDTA in the blood collection tube for plasma samples. Considering that EDTA and several KP metabolites function as a metal chelating agent, it is conceivable that EDTA and these metal chelating KP metabolites may bind or sequester together with the metals. Subsequently, this EDTA-KP metabolite-metal complexes might be removed during the isolation process. Among the KP metabolites, 3HK, AA, 3HAA, XA, QUIN and PIC have been demonstrated metal-chelating properties, with PIC having the capacity to chelate with the most number of metals.^3^ This finding could partially explain the higher PIC level observed in serum. In contrast, serum collection tube lacks EDTA which may allow for greater amount of PIC to remain available for detection.

The next aim of our study examined the stability of the KP metabolites in each blood matrix if the collected sample cannot be processed immediately. This was to mimic unforeseen delays in processing samples by the participating clinic or research laboratory. Hence, KP metabolites were analysed in each blood matrix after a 24 hours incubation at 4°C. The concentration of various KP and serotonin metabolites in blood samples was lower compared to immediate processing samples. This was the case in all 3 different analytical methods. Reduced metabolite concentrations could be due to the presence of a cellular component such as macrophages that remain metabolically active.^38^ Alternatively, blood is a relatively high redox matrix, which can readily oxidize KP metabolites.^29^ The quantification of KP metabolites using HPLC in plasma and serum showed a lower concentration after 24 hours whereas measurement using LC-MS/MS generally did not result in reduced KP concentrations. This is likely due to the difference in the protein precipitation step where TCA was used in HPLC while methanol was used in LC-MS/MS. The acidity of TCA could have resulted in oxidation of KP metabolites such as 3HK, 3HAA, KYNA and AA.^29^ It is noteworthy that the KP metabolites remained stable once they are processed immediately for storage. Trepci et al^39^ showed that multiple freeze-thaw cycles and storage for up to 24 hours at room temperature had minimal impact on the stability of all the KP metabolites except for 3HAA.

In conclusion, our study has demonstrated that the choice of blood matrix and the timing of sample processing significantly influence the stability of the KP metabolites. While the serum or plasma are frequently the preferred choice of matrix in both research and clinical studies, it is essential to the isolate these matrices from whole blood immediately after collection to obtain optimal analytical KP data. Specifically, metabolites such as 3HK and 3HAA exhibit time-dependent variations, potentially attributed to their redox properties or interactions with blood cellular components. These metabolites were of particular interest due to their neurotoxic and immune modulating properties.^3^ PIC is another metabolite that is susceptible to alterations with extended incubation period as its level increases over time. This could potentially be facilitated by the metabolic active of cellular components, notably the pericytes.^40^ This thus results in an overrepresentation of the KP. Overall, our study underscores the importance of maintaining a short processing window to minimize such effects as emphasized by Gegner et al.^41^

## Limitation

While the gender distribution in our cohort was relatively even, it is crucial to acknowledge that the size of the study cohort is relatively small. With the exception of one participant, all individuals are under the age of 49 and this may potentially have an impact on the KP activity as ageing is associated with increased TRP metabolism.^42^ Furthermore, it is worth noting that our participants did not undergo fasting or dietary/supplement restriction such as vitamin B. These factors, specifically fasting^43^ and dietary supplements,^44^ have been shown to influence the activity of KP enzymes. Finally, it is important to consider the potential impact of BMI on the KP, which is not in my selection criteria, particularly in the context of obesity.^45^ Therefore, the measurement presented in this study may not accurately represent the general population. Another important parameter not examined in this study is free TRP in various matrices. A previous study by Morgan and Badawy^46^ showed that frozen storage of serum for 24 hours or at 4°C at lowers free TRP at approximately 40% while increasing binding of TRP to albumin.

## Supplemental Material

sj-docx-1-try-10.1177_11786469231213521 – Supplemental material for Stability Studies of Kynurenine Pathway Metabolites in Blood Components Define Optimal Blood Processing Conditions Click here for additional data file. Supplemental material, sj-docx-1-try-10.1177_11786469231213521 for Stability Studies of Kynurenine Pathway Metabolites in Blood Components Define Optimal Blood Processing Conditions by Benjamin Heng, Ananda Staats Pires, Sharron Chow, Shivani Krishnamurthy, Brooke Bonnell, Sonia Bustamante and Gilles J Guillemin in International Journal of Tryptophan Research

## References

[1] Badawy AA. Kynurenine pathway of tryptophan metabolism: regulatory and functional aspects. Int J Tryptophan Res. 2017;10:1178646917691938. 28469468 10.1177/1178646917691938 PMC5398323

[2] Mándi Y Stone TW Guillemin GJ Vécsei L Williams RO. Editorial: multiple implications of the kynurenine pathway in inflammatory diseases: diagnostic and therapeutic applications. Front Immunol. 2022;13:860867. 35251052 10.3389/fimmu.2022.860867 PMC8892578

[bibr3-11786469231213521] Pires AS Sundaram G Heng B Krishnamurthy S Brew BJ Guillemin GJ. Recent advances in clinical trials targeting the kynurenine pathway. Pharmacol Ther. 2022;236:108055. 34929198 10.1016/j.pharmthera.2021.108055

[4] Platten M Nollen EAA Röhrig UF Fallarino F Opitz CA. Tryptophan metabolism as a common therapeutic target in cancer, neurodegeneration and beyond. Nat Rev Drug Discov. 2019;18:379-401. 30760888 10.1038/s41573-019-0016-5

[5] Tóth F Cseh EK Vécsei L. Natural molecules and neuroprotection: kynurenic acid, pantethine and α-lipoic acid. Int J Mol Sci. 2021;22:403. 33401674 10.3390/ijms22010403 PMC7795784

[6] Goldstein LE Leopold MC Huang X , et al. 3-hydroxykynurenine and 3-hydroxyanthranilic acid generate hydrogen peroxide and promote alpha-crystallin cross-linking by metal ion reduction. Biochemistry. 2000;39:7266-7275. 10852726 10.1021/bi992997s

[7] Guillemin GJ. Quinolinic acid: neurotoxicity. FEBS J. 2012;279:1355. 10.1111/j.1742-4658.2012.08493.x 22251552

[8] Guillemin GJ Williams KR Smith DG Smythe GA Croitoru-Lamoury J Brew BJ. Quinolinic acid in the pathogenesis of Alzheimer’s disease. Adv Exp Med Biol. 2003;527:167-176. 15206729 10.1007/978-1-4615-0135-0_19

[9] Guillemin GJ Brew BJ Noonan CE Takikawa O Cullen KM. Indoleamine 2,3 dioxygenase and quinolinic acid immunoreactivity in Alzheimer’s disease hippocampus. Neuropathol Appl Neurobiol. 2005;31:395-404. 16008823 10.1111/j.1365-2990.2005.00655.x

[10] Grant RS Coggan SE Smythe GA. The physiological action of picolinic acid in the human brain. Int J Tryptophan Res. 2009;2:71-79. 22084583 10.4137/ijtr.s2469 PMC3195224

[11] Taleb O Maammar M Klein C Maitre M Mensah-Nyagan AG. A role for xanthurenic acid in the control of brain dopaminergic activity. Int J Mol Sci. 2021;22:6974. 34203531 10.3390/ijms22136974 PMC8268472

[12] Taleb O Maammar M Brumaru D , et al. Xanthurenic acid binds to neuronal G-protein-coupled receptors that secondarily activate cationic channels in the cell line NCB-20. PLoS One. 2012;7:e48553. 10.1371/journal.pone.0048553 PMC3491036 23139790

[13] Yan J Han VX Heng B Guillemin GJ Bandodkar S Dale RC. Development of a translational inflammation panel for the quantification of cerebrospinal fluid Pterin, Tryptophan-Kynurenine and Nitric oxide pathway metabolites. EBioMedicine. 2022;77:103917. 35279631 10.1016/j.ebiom.2022.103917 PMC8914118

[bibr14-11786469231213521] Anesi A Rubert J Oluwagbemigun K , et al. Metabolic profiling of human plasma and urine, targeting tryptophan, tyrosine and branched chain amino acid pathways. Metabolites. 2019;9:261. 31683910 10.3390/metabo9110261 PMC6918267

[15] Sadok I Gamian A Staniszewska MM. Chromatographic analysis of tryptophan metabolites. J Sep Sci. 2017;40:3020-3045. 28590049 10.1002/jssc.201700184 PMC5575536

[16] Mrštná K Krčmová LK Švec F. Advances in kynurenine analysis. Clin Chim Acta. 2023;547:117441. 37321530 10.1016/j.cca.2023.117441

[17] Brown SJ Christofides K Weissleder C , et al. Sex- and suicide-specific alterations in the kynurenine pathway in the anterior cingulate cortex in major depression. Neuropsychopharmacology. Published online September 21, 2023. doi:10.1038/s41386-023-01736-8 PMC10789861 37735504

[18] Brundin L Sellgren CM Lim CK , et al. An enzyme in the kynurenine pathway that governs vulnerability to suicidal behavior by regulating excitotoxicity and neuroinflammation. Transl Psychiatry. 2016;6:e865. 10.1038/tp.2016.133 PMC5022080 27483383

[19] Myint AM Schwarz MJ Verkerk R , et al. Reversal of imbalance between kynurenic acid and 3-hydroxykynurenine by antipsychotics in medication-naïve and medication-free schizophrenic patients. Brain Behav Immun. 2011;25:1576-1581. 21620952 10.1016/j.bbi.2011.05.005

[20] Hare SM Adhikari BM Mo C , et al. Tryptophan challenge in individuals with schizophrenia and healthy controls: acute effects on circulating kynurenine and kynurenic acid, cognition and cerebral blood flow. Neuropsychopharmacology. 2023;48:1594-1601. 37118058 10.1038/s41386-023-01587-3 PMC10516920

[21] Yu Z Kastenmüller G He Y , et al. Differences between human plasma and serum metabolite profiles. PLoS One. 2011;6:e21230. 10.1371/journal.pone.0021230 PMC3132215 21760889

[22] Panuwet P Hunter RE Jr D’Souza PE , et al. Biological matrix effects in quantitative tandem mass spectrometry-based analytical methods: advancing biomonitoring. Crit Rev Anal Chem. 2016;46:93-105. 25562585 10.1080/10408347.2014.980775 PMC4695332

[23] Matuszewski BK Constanzer ML Chavez-Eng CM. Strategies for the assessment of matrix effect in quantitative bioanalytical methods based on HPLC-MS/MS. Anal Chem. 2003;75:3019-3030. 12964746 10.1021/ac020361s

[24] Plebani M. Quality indicators to detect pre-analytical errors in laboratory testing. Clin Biochem Rev. 2012;33:85-88. 22930602 PMC3428256

[25] Yin P Lehmann R Xu G. Effects of pre-analytical processes on blood samples used in metabolomics studies. Anal Bioanal Chem. 2015;407:4879-4892. 25736245 10.1007/s00216-015-8565-x PMC4471316

[26] Staats Pires A Heng B Tan VX , et al. Kynurenine, tetrahydrobiopterin, and cytokine inflammatory biomarkers in individuals affected by diabetic neuropathic pain. Front Neurosci. 2020;14:890. 32973438 10.3389/fnins.2020.00890 PMC7472959

[27] Bustamante S Yau Y Boys V , et al. Tryptophan metabolism ‘Hub’ gene expression associates with increased inflammation and severe disease outcomes in COVID-19 infection and inflammatory bowel disease. Int J Mol Sci. 2022;23:14776. 36499104 10.3390/ijms232314776 PMC9737535

[28] Serkova NJ Standiford TJ Stringer KA. The emerging field of quantitative blood metabolomics for biomarker discovery in critical illnesses. Am J Respir Crit Care Med. 2011;184:647-655. 21680948 10.1164/rccm.201103-0474CI PMC3208597

[29] Darlington LG Forrest CM Mackay GM , et al. On the biological importance of the 3-hydroxyanthranilic acid: anthranilic acid ratio. Int J Tryptophan Res. 2010;3:51-59. 22084587 10.4137/ijtr.s4282 PMC3195249

[30] Kronenberg F Trenkwalder E Kronenberg MF König P Utermann G Dieplinger H. Influence of hematocrit on the measurement of lipoproteins demonstrated by the example of lipoprotein(a). Kidney Int. 1998;54:1385-1389. 9767560 10.1046/j.1523-1755.1998.00086.x

[31] Fic E Kedracka-Krok S Jankowska U Pirog A Dziedzicka-Wasylewska M. Comparison of protein precipitation methods for various rat brain structures prior to proteomic analysis. Electrophoresis. 2010;31:3573-3579. 20967768 10.1002/elps.201000197

[32] Rajalingam D Loftis C Xu JJ Kumar TK. Trichloroacetic acid-induced protein precipitation involves the reversible association of a stable partially structured intermediate. Protein Sci. 2009;18:980-993. 19388015 10.1002/pro.108 PMC2771300

[33] Cangiano C Cardelli P Peverini P , et al. Effect of kynurenine on tryptophan-albumin binding in human plasma. Adv Exp Med Biol. 1999;467:279-282. 10721066 10.1007/978-1-4615-4709-9_35

[34] Stringer KA Younger JG McHugh C , et al. Whole blood reveals more metabolic detail of the human metabolome than serum as measured by 1H-NMR spectroscopy: implications for sepsis metabolomics. Shock. 2015;44:200-208. 26009817 10.1097/SHK.0000000000000406 PMC4537695

[35] Emwas AH. The strengths and weaknesses of NMR spectroscopy and mass spectrometry with particular focus on metabolomics research. Methods Mol Biol. 2015;1277:161-193. 25677154 10.1007/978-1-4939-2377-9_13

[36] Schrimpe-Rutledge AC Codreanu SG Sherrod SD McLean JA. Untargeted metabolomics strategies-challenges and emerging directions. J Am Soc Mass Spectrom. 2016;27:1897-1905. 27624161 10.1007/s13361-016-1469-y PMC5110944

[37] Roux A Lison D Junot C Heilier JF. Applications of liquid chromatography coupled to mass spectrometry-based metabolomics in clinical chemistry and toxicology: a review. Clin Biochem. 2011;44:119-135. 20800591 10.1016/j.clinbiochem.2010.08.016

[38] Breier M Wahl S Prehn C , et al. Targeted metabolomics identifies reliable and stable metabolites in human serum and plasma samples. PLoS One. 2014;9: e89728. 10.1371/journal.pone.0089728 PMC3933650 24586991

[39] Trepci A Imbeault S Wyckelsma VL , et al. Quantification of plasma kynurenine metabolites following one bout of sprint interval exercise. Int J Tryptophan Res. 2020;13:1178646920978241. 33354112 10.1177/1178646920978241 PMC7734489

[40] Owe-Young R Webster NL Mukhtar M , et al. Kynurenine pathway metabolism in human blood-brain-barrier cells: implications for immune tolerance and neurotoxicity. J Neurochem. 2008;105:1346-1357. 18221377 10.1111/j.1471-4159.2008.05241.x

[41] Gegner HM Naake T Dugourd A , et al. Pre-analytical processing of plasma and serum samples for combined proteome and metabolome analysis. Front Mol Biosci. 2022;9:961448. 36605986 10.3389/fmolb.2022.961448 PMC9808085

[42] Sorgdrager FJH Vermeiren Y Van Faassen M , et al. Age- and disease-specific changes of the kynurenine pathway in Parkinson’s and Alzheimer’s disease. J Neurochem. 2019;151:656-668. 31376341 10.1111/jnc.14843 PMC6899862

[43] Solianik R Schwieler L Trepci A Erhardt S Brazaitis M. Two-day fasting affects kynurenine pathway with additional modulation of short-term whole-body cooling: a quasi-randomised crossover trial. Br J Nutr. 2023;129: 992-999. 10.1017/S0007114522002069 35791050

[44] Ciorba MA. Kynurenine pathway metabolites: relevant to vitamin B-6 deficiency and beyond. Am J Clin Nutr. 2013;98:863-864. 23985806 10.3945/ajcn.113.072025 PMC4498264

[45] Farup PG Hamarsland H Mølmen KS Ellefsen S Hestad K. The kynurenine pathway in healthy subjects and subjects with obesity, depression and chronic obstructive pulmonary disease. Pharmaceuticals (Basel). 2023;16:351. 36986451 10.3390/ph16030351 PMC10053928

[46] Morgan CJ Badawy AA. Effects of storage on binding and stability of tryptophan in human serum. Ann Clin Biochem. 1994;31(Pt 2):190-192. 8060100 10.1177/000456329403100215

