# Supplementary material for: Stability Studies of Kynurenine Pathway Metabolites in Blood Components Define Optimal Blood Processing Conditions
Source: Int J Tryptophan Res. 2023 Dec 15;16:11786469231213521. doi: 10.1177/11786469231213521 (PMC10725091; doi:10.1177/11786469231213521)
Supplement: sj-docx-1-try-10.1177_11786469231213521 – Supplemental material for Stability Studies of Kynurenine Pathway Metabolites in Blood Components Define Optimal Blood Processing Conditions [file sj-docx-1-try-10.1177_11786469231213521.docx]

SUPPLEMENTARY TABLES

Title: Stability Studies of Kynurenine Pathway Metabolites in Blood Components Define Optimal Blood Processing Conditions

Benjamin Heng^1^*^#^, Ananda Staats Pires^1^*, Sharron Chow^1^, Shivani Krishnamurthy^1^, Brooke Bonnell^1^, David B. Lovejoy^1^, Sonia Bustamante^2+^ and Gilles J. Guillemin^1+^

^1^ Macquarie Medical School, Faculty of Medicine and Health Sciences, Macquarie University, Sydney, Australia

^2^ Bioanalytical Mass Spectrometry Facility, University of New South Wales, Sydney, Australia

* Co-first author; ^+^ Co-last author, ^#^ Corresponding author

##### **Supplementary table S1: Kynurenine pathway metabolite concentrations measured by HPLC and uHPLC in whole blood, plasma and serum from healthy volunteers at day 1 and day 2.**

| Metabolite | Day 1 | | | | | | Day 2 | | | | | | 2-Way ANOVA | | |
| --- | --- | --- | --- | --- | --- | --- | --- | --- | --- | --- | --- | --- | --- | --- | --- |
|  | **Whole blood (n=12)** | | **Plasma (n=12)** | | **Serum (n=12)** | | **Whole blood (n=12)** | | **Plasma (n=12)** | | **Serum (n=12)** | | ***adj. p-value* (Time)** | ***adj. p-value* (Matrix)** | ***adj. p-value* (Time-Matrix)** |
|  | **Mean** | **SD** | **Mean** | **SD** | **Mean** | **SD** | **Mean** | **SD** | **Mean** | **SD** | **Mean** | **SD** |  |  |  |
| TRP µM | 16.6 | 7.1 | 40.6 | 14.6 | 43.6 | 17 | 14.9 | 7.3 | 45 | 11.6 | 47 | 11.9 | .3091 | <.001 | .4078 |
| KYN µM | 0.9 | 0.2 | 1.3 | 0.2 | 1.3 | 0.2 | 0.8 | 0.1 | 1.3 | 0.2 | 1.3 | 0.2 | .0105 | <.001 | .1653 |
| KYNA nM | 8.4 | 6.2 | 28.6 | 8.5 | 29.9 | 7.2 | 10.3 | 5 | 24.3 | 9.3 | 22.5 | 5.7 | .0059 | .0059 | <.001 |
| 3HK nM | 18.3 | 8.6 | 44.7 | 36.9 | 57.6 | 48 | 16.3 | 7.2 | 27.5 | 17.5 | 38.1 | 28.4 | .0071 | .0223 | .2274 |
| 3HAA nM | 5.4 | 2.2 | 12.3 | 7.5 | 17.9 | 7.4 | 4.5 | 2.3 | 7.5 | 3 | 14.8 | 5.2 | <.001 | <.001 | .0732 |
| AA nM | 31.6 | 16.1 | 24.1 | 9.1 | 27.8 | 13 | 26.5 | 17.4 | 21.4 | 4.5 | 27.6 | 11.6 | .0491 | .4990 | .5071 |
| Abbreviations: KYN: Kynurenine; TRP: Tryptophan; KYNA: Kynurenic acid; 3HK: 3-hydroxykynurenine; 3HAA: 3-hydroxyanthranilic acid; AA: Anthranilic acid. | | | | | | | | | | | | | | | |

##### **Supplementary table S2: Kynurenine pathway metabolite concentrations measured by GC/MS in whole blood, plasma and serum from healthy volunteers at day 1 and day 2.**

| Metabolite | Day 1 | | | | | | Day 2 | | | | | | 2-Way ANOVA | | | |
| --- | --- | --- | --- | --- | --- | --- | --- | --- | --- | --- | --- | --- | --- | --- | --- | --- |
|  | **Whole blood (n=12)** | | **Plasma (n=12)** | | **Serum (n=12)** | | **Whole blood (n=12)** | | **Plasma (n=12)** | | **Serum (n=12)** | | ***adj. p-value* (Time)** | ***adj. p-value* (Matrix)** | ***adj. p-value* (Time-Matrix)** |  |
|  | **Mean** | **SD** | **Mean** | **SD** | **Mean** | **SD** | **Mean** | **SD** | **Mean** | **SD** | **Mean** | **SD** |  |  |  |  |
| PIC nM | 178.2 | 68.4 | 240.6 | 95.8 | 361.1 | 125.3 | 222.7 | 64.6 | 281 | 90.3 | 419.3 | 136 | <.001 | <.001 | .8594 |  |
| QUIN nM | 617.3 | 330.8 | 832.8 | 420.7 | 781.6 | 423.5 | 518 | 218.7 | 937 | 627.3 | 864.3 | 611.5 | .8093 | .0749 | .7228 |  |
| Abbreviations: PIC: Picolinic acid; QUIN: Quinolinic acid. | | | | | | | | | | | | | | | |  |

##### **Supplementary table S3: Kynurenine pathway metabolite concentrations measured by LC-MS/MS in whole blood, plasma and serum from healthy volunteers at day 1 and day 2.**

| Metabolite | Day 1 | | | | | | Day 2 | | | | | | 2-Way ANOVA | | | |
| --- | --- | --- | --- | --- | --- | --- | --- | --- | --- | --- | --- | --- | --- | --- | --- | --- |
|  | **Whole blood (n=12)** | | **Plasma (n=12)** | | **Serum (n=12)** | | **Whole blood (n=12)** | | **Plasma (n=12)** | | **Serum (n=12)** | **Whole blood (n=12)** | ***adj. p-value* (Time)** | ***adj. p-value* (Matrix)** | ***adj. p-value* (Time-Matrix)** |  |
|  | **Mean** | **SD** | **Mean** | **SD** | **Mean** | **SD** | **Mean** | **SD** | **Mean** | **SD** | **Mean** | **SD** |  |  |  |  |
| TRP µM | 34.6 | 15.2 | 52.9 | 21.6 | 52.4 | 25.7 | 31 | 14.3 | 50.7 | 24.5 | 58.8 | 24.3 | .8927 | <.05 | .0556 |  |
| KYN µM | 0.9 | 0.3 | 1.4 | 0.4 | 1.3 | 0.5 | 0.8 | 0.2 | 1.2 | 0.4 | 1.5 | 0.5 | .3281 | <.001 | <.05 |  |
| KYNA nM | 2.6 | 0.8 | 4.5 | 2 | 4.1 | 2.1 | 2.2 | 0.7 | 4.1 | 2.1 | 4.5 | 2.2 | .5990 | <.01 | .2060 |  |
| 3HK nM | 4.3 | 2.9 | 26 | 11.7 | 25.6 | 5.7 | 1.5 | 1.8 | 23.5 | 15.3 | 27.2 | 8.6 | .0357 | <.001 | <.05 |  |
| 3HAA nM | 6.6 | 5.2 | 23.2 | 12.7 | 22.5 | 16.6 | 4 | 4.1 | 17.1 | 10.1 | 25.8 | 15.6 | .1292 | <.001 | <.05 |  |
| AA nM | 7.6 | 4 | 5.9 | 2.2 | 10.6 | 5.2 | 5.9 | 2.8 | 6.9 | 3.5 | 10.5 | 5.7 | .5841 | <.05 | .0949 |  |
| XA nM | 12.5 | 5.8 | 31.4 | 18.3 | 28.9 | 19 | 11.8 | 6.2 | 29.1 | 18 | 30.9 | 15.4 | .7041 | <.05 | .1820 |  |
| Abbreviations: KYN: Kynurenine; TRP: Tryptophan; KYNA: Kynurenic acid; 3HK: 3-hydroxykynurenine; 3HAA: 3-hydroxyanthranilic acid; AA: Anthranilic acid; XA: Xanthurenic acid. | | | | | | | | | | | | | | | |  |

##### **Supplementary table S4: Serotonin-related metabolite concentrations measured by LC-MS/MS in whole blood, plasma and serum from healthy volunteers at day 1 and day 2.**

| Metabolite | Day 1 | | | | | | Day 2 | | | | | | 2-Way ANOVA | | |
| --- | --- | --- | --- | --- | --- | --- | --- | --- | --- | --- | --- | --- | --- | --- | --- |
|  | **Whole blood (n=12)** | | **Plasma (n=12)** | | **Serum (n=12)** | | **Whole blood (n=12)** | | **Plasma (n=12)** | | **Serum (n=12)** | | ***adj. p-value* (Time)** | ***adj. p-value* (Matrix)** | ***adj. p-value* (Time-Matrix)** |
|  | **Mean** | **SD** | **Mean** | **SD** | **Mean** | **SD** | **Mean** | **SD** | **Mean** | **SD** | **Mean** | **SD** |  |  |  |
| Serotonin nM | 256.2 | 202.8 | 346.4 | 256.6 | 457.7 | 386 | 195.5 | 130.8 | 305.6 | 299.8 | 621.8 | 440.6 | .8704 | .0505 | <.01 |
| 5HT µM | 3.2 | 1.2 | 4.6 | 1.9 | 5.6 | 3.1 | 2.7 | 1.3 | 5.2 | 3 | 6.5 | 2.7 | .2955 | <.01 | .1639 |
| 5IHAA nM | 13 | 11.9 | 24.2 | 12.3 | 31.6 | 15.7 | 18.7 | 13.4 | 30.2 | 15.4 | 43 | 19.5 | <.001 | <.01 | .3816 |
| Abbreviations: 5HT: 5-hydroxytryptophan; 5HIAA: 5-hydroxyindoleacetic acid. | | | | | | | | | | | | | | | |
